# Total small bowel resection in a patient who refused surgery: why did the surgeon do it?^†^

**DOI:** 10.1093/jscr/rjae765

**Published:** 2024-12-12

**Authors:** Reynold Henry, Patrick McGillen, Wesley E Barry, Julie S Wecsler, Brandon Knight, Kazuhide Matsushima, Carin Van Zyl, Peter Crookes

**Affiliations:** Department of Surgery, University of Southern California, 1500 San Pablo St, Los Angeles, CA 90033, United States; Department of Surgery, University of Southern California, 1500 San Pablo St, Los Angeles, CA 90033, United States; Department of Surgery, University of Southern California, 1500 San Pablo St, Los Angeles, CA 90033, United States; Department of Surgery, University of Southern California, 1500 San Pablo St, Los Angeles, CA 90033, United States; Department of Surgery, University of Southern California, 1500 San Pablo St, Los Angeles, CA 90033, United States; Department of Surgery, University of Southern California, 1500 San Pablo St, Los Angeles, CA 90033, United States; Department of Palliative Medicine, University of Southern California, 1500 San Pablo St, Los Angeles, CA 90033, United States; Department of Surgery, University of Southern California, 1500 San Pablo St, Los Angeles, CA 90033, United States

**Keywords:** medical ethics, Roux-en-Y gastric bypass, autonomy, beneficence

## Abstract

Gastric surgery may result in internal herniation of bowel, weeks to years after the initial surgery and can result in rapid onset of death if not promptly treated. We present a case in which a patient with this complication underwent surgery despite his clear refusal of surgery. The patient had a remote history of gastrectomy for malignancy. Several years later, he presented with the feared complication of an internal herniation to a local hospital. The initial surgeon recommended urgent operation to correct this. However, the patient refused. When he became comatose, the surgeon performed surgery, but the patient’s condition continued to deteriorate. The surgeon performed further surgery, eventually removing the entire small bowel, leaving nothing connecting the esophagus or effluent from the liver and pancreas. Although this was a fatal situation, he had temporary recovery sufficient to understand the nature of his prognosis and survived several weeks.

## Introduction

Every surgeon embarks on the same goals before an operation; to save a life or improve said life. To achieve this, we hone our expertise through years of training, planning and recall lessons from prior mistakes. As a result, ˂20% of the 230 million patients undergoing an operation each year will experience a significant surgical complication and ˂1% will result in a mortality [1]. But despite our best-efforts, complications arise, and poor outcomes occur.

As surgeons, we struggle with the more puzzling cases in which an operation presents new risk for a patient’s life that they are unwilling to understand or accept. Our life saving goals can sometimes be pitted against our patient’s own autonomy. Physician-patient conflict and poor outcomes are often a result of our failure to balance the four basic ethical principles we uphold as physicians—autonomy, beneficence, nonmaleficence, and justice. We present the following case in which a surgical complication and subsequent treatment highlights the ethical challenges in balancing these principles.

## Case presentation

**Figure 1:**
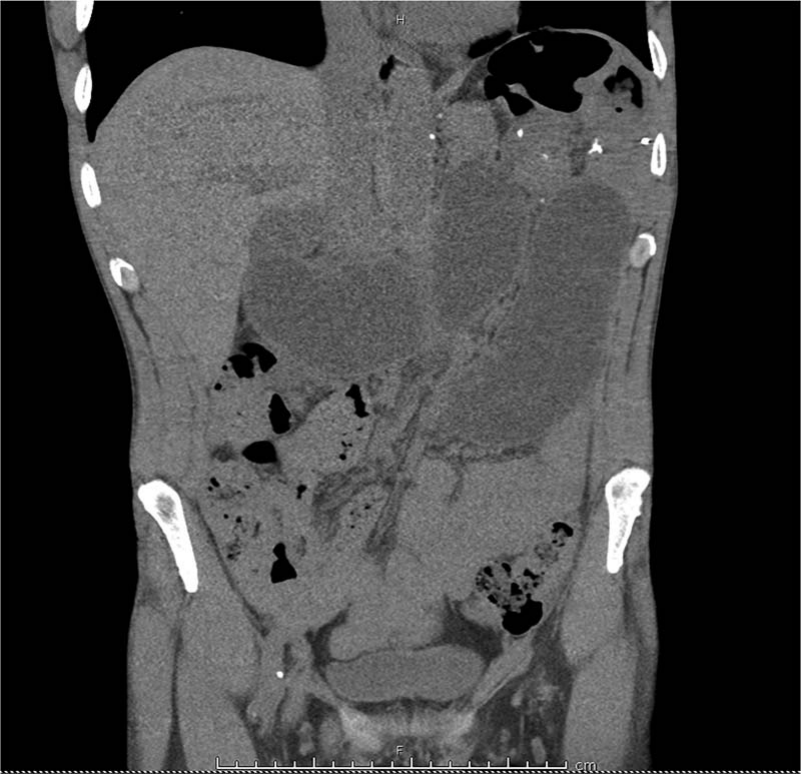
A CT scan of the patient’s abdomen demonstrating obstruction suggestive of internal hernia.

**Figure 2:**
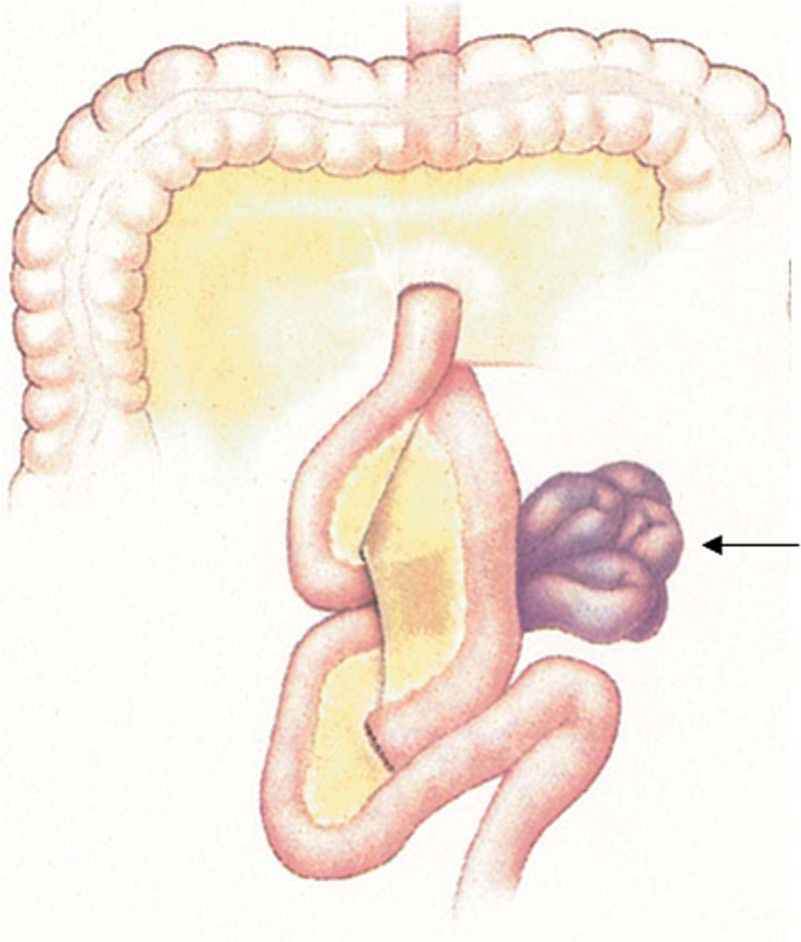
A conceptualization of an internal hernia after Roux-en-Y reconstruction. Permission obtained from Peter M. van Brussel, MD, PhD.

Mr R was a 55-year-old male who presented to a local hospital with abdominal pain 10 years after radical total gastrectomy for adenocarcinoma with Roux-en-Y reconstruction. Computed tomography (CT) showed a small bowel internal hernia (IH) with evidence suggestive of bowel ischemia (Fig. 1). A small bowel IH is a rare but dreaded complication following Roux-en-Y in which a segment of small bowel protrudes through a defect in the peritoneum or mesentery (Fig. 2). IH is thought to occur in ˂5% of cases, though prompt diagnosis and surgery of IH are essential as delayed treatment may lead to bowel strangulation and perforation.

After identifying the IH with probable ongoing bowel ischemia, the patient was admitted to the hospital’s surgical team and emergent surgical exploration and resection of nonviable bowel was offered. After discussing the risks and benefits of surgery, including death if not surgically corrected, the patient declined surgery, much to the dismay of the operating team. The team’s note reflects a high degree of concern that the patient did not fully appreciate the severity of his condition and consequences of deferring surgery given his otherwise reasonable demeanor. Respecting the patient’s choice, the team continued with supportive care for IH until he predictably developed sepsis and respiratory failure necessitating intubation and aggressive intensive care resuscitation.

**Figure 3:**
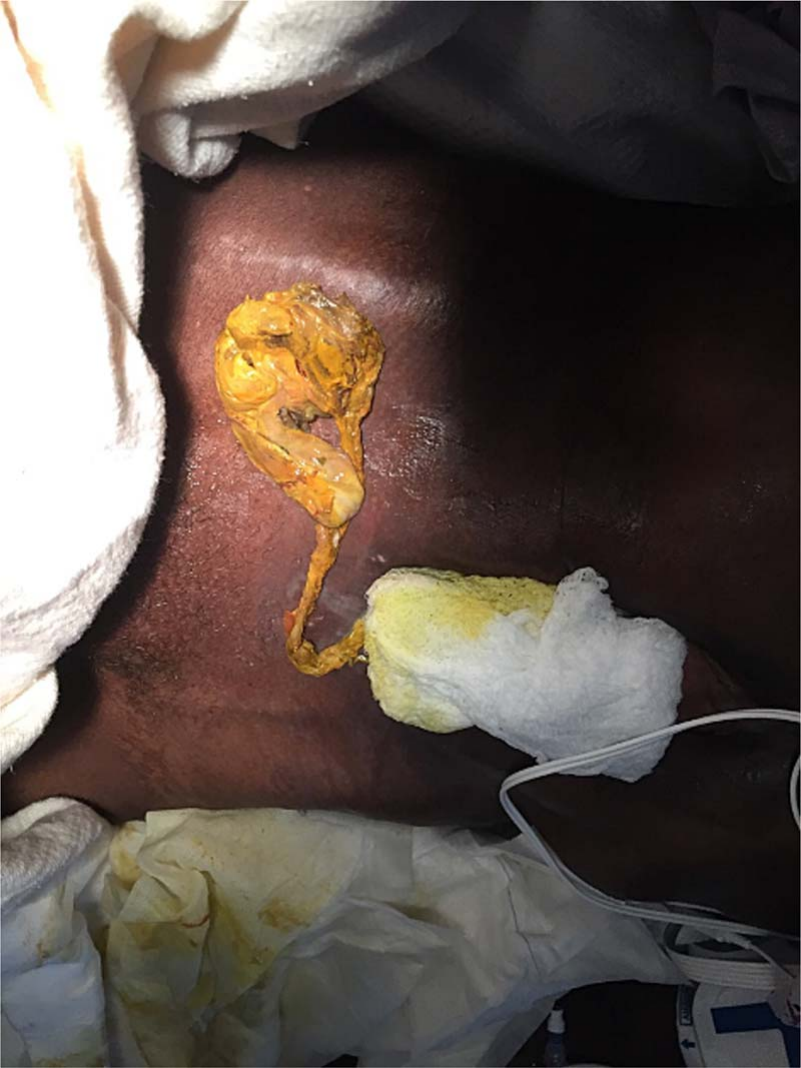
A picture of what remained of the patient’s cecum, completely detached from the small bowel.

Given the patient was no longer able to advocate for his own medical care, his surrogate decision maker, his sister, was informed of the dire circumstances. Without previous knowledge of his decision, she consented to surgery. Over the course of two operations the patient’s entire small bowel was resected after it was determined to be nonviable—a terminal diagnosis. The patient’s open-ended esophagus, bile duct, and pancreatic duct were left to drain directly into the abdominal cavity which was now covered by a temporary vacuum closure device (Fig. 3). Over the course of two weeks the patient developed renal failure requiring continuous hemodialysis and the endotracheal tube was replaced with a tracheotomy. Shortly thereafter, the patient was transferred to the surgery team of our hospital for possible small bowel transplant, a rare intervention.

Eventually the patient was able to breathe, respond to commands, and weaned from the ventilator. Though still requiring daily abdominal vacuum changes as result of the continuous flow of saliva, biliopancreatic fluid into the abdomen, he recovered his ability to communicate. He was not a candidate for small bowel transplant. He understood his fatal prognosis and signed a do not resuscitate (DNR) order. During the remainder of his life in the hospital he said goodbye to loved ones and survived long enough to enjoy a piece of cake on his final birthday. A few days later he developed a lethal arrhythmia and passed away.

## Discussion

Contemporary bioethics are framed in terms derived from the landmark *Principles of Biomedical Ethics*, first published 40 years ago. The text distills the ethical guidance of physician-patient interaction into four key principles: autonomy, benefice, nonmaleficence, and justice [2]. We recognize autonomy as a patient’s right to self-determination which ethically enjoins us to obtain consent prior to any operation. Autonomy is made up of three key components: understanding, intentionality, and voluntariness [3]. The ethical obligation to provide a patient with information to make informed and reasonable decisions about their treatment has been formalized into a legal duty we know as “informed consent.” As a result of this obligation, we advocate better for patients. It compels us to provide a patient with necessary information and facilitate understanding to weigh the benefit against the harm without compromising their autonomy.

Mr R was informed of the dire circumstance of his untreated IH and his inevitable death unless surgical correction was attempted. He understood his choice to forego surgery and accepted that he would die as result. The concept of ‘informed refusal’ is now well established as an expression of autonomy. As surgeons, we struggle to accept this, particularly in circumstances where the potential benefits of an operation so grossly outweigh the risks, namely death [4]. Rather than accept his decision, the operative team questioned his capacity, ascribing his choice as an act of fatal misjudgment.

The operating team was legally bound to withhold lifesaving surgery, yet the potential to save a life dictates us to treat. Courts cases and textbooks provide the framework to navigate these circumstances, but they do not capture the intricacy, nuance, and intimacy of the relationships with our patients. If left with an ethical framework alone, it’s clear that an ethical breach of practice was made by ignoring Mr R’s autonomy. It is important to recognize in this case that a patient’s ability to understand our treatment does not beget the patient’s willingness to accept that treatment. To disagree does not implicate a patient’s incapacity or misunderstanding. Yet, Mr R enjoyed his final weeks of life pain-free and enjoying meaningful farewells and a final birthday because of a surgeon’s choice to weigh benefice over autonomy. Only Mr R can judge whether his extended life was adequate restitution for the autonomy he was denied.
